# Sustainable Agronomical Practices Affect Essential Oil Composition of *Tanacetum balsamita* L.

**DOI:** 10.3390/plants14152406

**Published:** 2025-08-03

**Authors:** Martina Grattacaso, Alessandra Bonetti, Sara Di Lonardo, Luigi Paolo D’Acqui

**Affiliations:** 1 Research Institute on Terrestrial Ecosystems (IRET), National Research Council (CNR), Via Madonna del Piano 10, 50019 Sesto Fiorentino, Italy; martina.grattacaso@iret.cnr.it (M.G.); alessandra.bonetti@cnr.it (A.B.); luigipaolo.dacqui@cnr.it (L.P.D.); 2 National Biodiversity Future Center (NBFC), Piazza Marina 61, 90133 Palermo, Italy

**Keywords:** *Tanacetum balsamita* L., essential oils, bioinoculants, compost, sustainable agricultural practices, gas chromatography

## Abstract

This study evaluated the influence of compost and bioinoculants (mycorrhizal fungi and plant growth-promoting bacteria) on the yield and composition of essential oil extracted from *Tanacetum balsamita* L. over two growing seasons. The plants were cultivated under four treatments: compost, bioinoculants, a combination (bioinoculants + compost), and a control. At each harvest, essential oil was extracted from fresh leaves via stem-flow distillation and analyzed using gas chromatography coupled with single quadrupole mass spectrometry. Twenty to twenty-four compounds were identified. Based on the dominant terpene derivative, the results indicated that *Tanacetum balsamita* L. cultivated in Italy belongs to “camphor” chemotype, a pharmacologically active compound known for its antimicrobial, anti-inflammatory, and analgesic properties. Moreover, three compounds, α-, β-phellandrene and myrtenol, were identified as typical of *Tanacetum balsamita* L. cultivated in Italy. Treatment effects were significant for some compounds (camphor, borneol, terpinen-4-ol, α-terpineol, dehydro sabinene ketone, and 3-thujanol), and the interaction between treatment and year was significant for a few compounds (borneol, terpinen-4-ol, dehydro sabinene ketone, 1,8-cineol, and 3-thujanol). These results emphasize the need to account for seasonal variation and underline the necessity of a deeper understanding of how experimental factors interact with them, especially in long-term essential oil studies.

## 1. Introduction

The search for sustainable practices that yield results comparable to high-intensity farming continues to cope with the effects of climate change while reducing environmental costs [1]. Following the transformative changes brought about via the Green Revolution—which emphasized the use of synthetic fertilizers, chemical pesticides, and high-yield crop varieties to dramatically boost agricultural productivity in the mid-20th century—there is now increasing global attention toward a new paradigm often referred to as the “microbial revolution” [2]. This emerging approach focuses on harnessing the potential of plant-associated microbial communities (also known as the plant microbiota) to promote plant health, increase crop yields, and reduce reliance on chemical inputs. The microbial revolution advocates for the sustainable use and targeted manipulation of beneficial microbes—such as plant growth-promoting rhizobacteria, endophytes, and mycorrhizal fungi—that naturally inhabit plant roots, leaves, and surrounding soil. These microbes play essential roles in nutrient acquisition, pathogen resistance, stress tolerance, and metabolic enhancement. Recent advances in microbial ecology, genomics, and biotechnology have made it increasingly feasible to isolate, identify, and apply specific microbial strains or microbial consortia to crops as biofertilizers, biostimulants, or biopesticides. This strategy is gaining particular interest not only for staple crops but also for aromatic and medicinal plants, where microbial associations may influence not just growth but also the biosynthesis of essential oils and other bioactive compounds. Thus, the shift toward a microbial revolution represents a sustainable and innovative trajectory for modern agriculture, aiming to complement or even reduce the need for traditional agrochemicals while improving plant productivity and resilience across diverse agricultural systems, including aromatic plant cultivation [3,4,5,6]. Plant growth-promoting rhizobacteria (PGPR), including *Bacillus*, *Paenibacillus*, *Azospirillum*, and *Azotobacter*, play a key role in this approach. These microorganisms contribute to nitrogen fixation, phosphorus solubilization, siderophore production, phytohormone synthesis, and biological control. Over the past decade, many formulations have been applied to various crops to harness these benefits [3,7]. Similarly, arbuscular mycorrhizal fungi (AMF), belonging to the subphylum *Glomeromycotina*, form mutualistic symbiosis with most land plants. In exchange for carbon derived from photosynthesis, AMF enhance water, phosphorus, and nitrogen uptake from plants, improving plant resilience to biotic and abiotic stresses [8]. The improved plant nutritional status induced via beneficial microorganisms has been associated with increased secondary metabolite production and enhanced yield in several crops and aromatics [3,9,10,11,12,13]. Degraded soil, characterized by poor aggregation, high bulk density, low porosity, and slow water infiltration, is a major environmental constraint limiting plant growth and productivity [14]. Various organic amendments, such as manures, have been studied for their effectiveness in soil remediation [15]. Long-term compost application (20 years at an annual rate of 15 t ha^−1^) has been shown to increase humus content by 0.4–0.5%. Beyond improving crop yield, organic amendments could also enhance the production of bioactive compounds in plants used in pharmaceutical, cosmetic, and nutraceutical industries. Among the numerous types of composts available, green compost, primarily derived from organic urban and agro-industrial waste, presents a sustainable alternative with the lowest net flux of greenhouse gases compared with other waste treatment options [16]. Its agronomic use is recognized as an effective recycling strategy to mitigate increasing waste production in Europe [17,18]. Green compost is widely used in both intensive and extensive agricultural systems, as well as for environmental restoration and home gardening. It enhances soil chemical, physical, and biological properties by gradually releasing nutrients through organic matter mineralization [17,19]. The effect of green compost on the plant–soil system depends on several factors, including its chemical composition, soil type, crop species, climate, and ecological conditions. Studies have shown that applying over 30 Mg ha^−1^ of mature green compost under different conditions significantly increases soil organic carbon (SOC), the C/N ratio, total nitrogen (TN), and soil pH [20,21,22,23]. Furthermore, compost has been identified as an effective solution for rehabilitating saline soils by enhancing nutrient availability and microbial activity [15,17,19,24,25].

**Figure 1:**
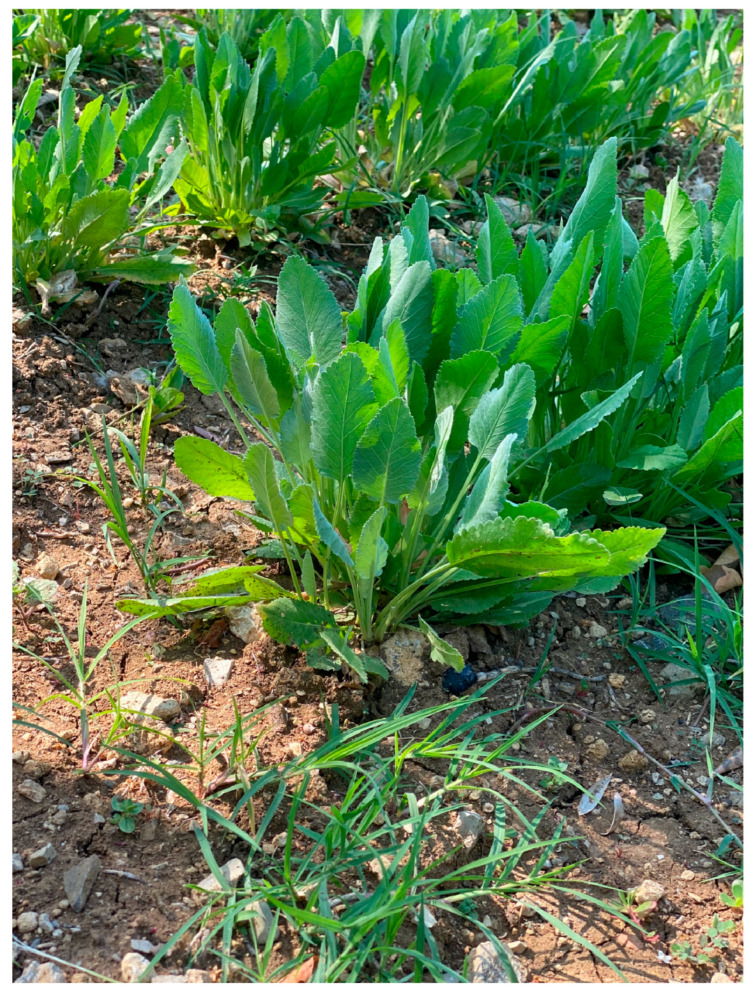
A particular of *Tanacetum balsamita* L. cultivation in Italy.

*Tanacetum balsamita* L. (Linnaeus, 1753), commonly known as costmary (Figure 1), has been valued for its medicinal properties since ancient times, and it is used by Egyptians, Greeks, and Romans. The name “balsamita” or “balsam herb” derives from the plant’s pleasant, aromatic, balsamic scent produced via volatile oils stored in leaf glands.

Originally native to Asia Minor, Asia, and Australia, costmary has become naturalized in many parts of eastern and southern Europe, as well as Turkey [26], where it is traditionally used for its antimicrobial properties [27]. Previous studies on costmary essential oils from Lithuania, Romania, Poland, Germany, and Russia have identified carvone and camphor as the main constituents, with concentrations ranging from 9.2 to 70.0% and from 0 to 91.0%, respectively [28,29,30]. Based on the dominant terpene derivative, four chemotypes of costmary essential oil have been identified: camphor-type [29], carvone-type [29], camphor/thujone-type [31], a carvone-chemotype producing considerable amounts of α-thujone [30]. Moreover, recent GC-MS works about the employment of essential oil components, such as carvone and camphor, integrated with a morphological analysis were employed to classify different species of *Tanacetum* [31]. Costmary leaves have been traditionally used in ethnomedicine for their hepatoprotective, tonic, sedative, pain relief, and astringent properties [32]. The essential oil and plant extracts have demonstrated analgesic, anti-inflammatory, antimicrobial, and antioxidant activities, supporting their traditional use in treating inflammatory conditions [33,34,35,36]. Refs. [32,36] reviewed costmary’s phytochemical profile and applications, highlighting its potential as a “forgotten plant” with promising uses in functional food development, as recently demonstrated also by [37].

Currently, to our knowledge, there is a limited understanding of how sustainable agronomic inputs (compost and bioinoculants) influence the essential oil yield and composition in aromatic species, in particular in *Tanacetum balsamita* L., over multiple growing seasons. This study aimed to evaluate the effects of compost, bioinoculants, and their combination on EOs synthesis in costmary plants cultivated in central Italy. We hypothesized that the application of compost, bioinoculants, or their combination would significantly modify the yield and chemical profile of essential oils in *Tanacetum balsamita* L., with effects across the two studied growing seasons.

## 2. Results and Discussion

**Figure 2:**
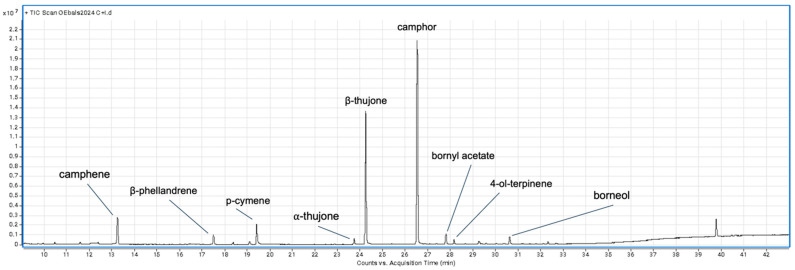
GC chromatogram of essential oils from fresh leaves of *Tanacetum balsamita* L.

A typical chromatogram of essential oil extracted from *Tanacetum balsamita* L. is shown in Figure 2.

In total, twenty molecules were identified in 2023 and twenty-four in 2024 (Table 1), primarily comprising monoterpenes and terpenes. In 2023, the major components included borneol, camphor, 1,8-cineol, α-thujone, and terpinene 4-ol. In 2024, these were supplemented with α-phellandrene and β-thujone, among the most abundant constituents. While the total concentration of essential oils remained unchanged between 2023 and 2024 (5.03 ± 0.34 mg g FW^−1^ and 4.23 ± 0.60 mg g FW^−1^, respectively), the composition and relative abundance of individual compounds varied. Additionally, no changes in chemical composition were observed when chromatograms from *T. balsamita* that had been subjected to different treatments were compared; however, notable differences in the relative abundance of several compounds were observed, suggesting treatment-dependent metabolic modulation. This compositional variability could be linked to the plant’s phenotypic plasticity, through which metabolic pathways adjust dynamically in response to external inputs such as organic amendments and microbial bioeffectors. Variable essential oil composition in tansy (*Tanacetum vulgare* L.) was observed even in [38]. In this paper, the authors suggest that the high variability in the composition of essential oils could be related to the high adaptability of this species to the environment. A similar trend was reported by [39] in the essential oil composition of sweet marjoram (*Marjorana hortensis* L.) cultivated with compost and biofertilizers.

Then, for each compound, some differences were found between the two growing seasons, 2023 and 2024 (Table 1), as was already stated also by other authors for other, different aromatic species [40].

Camphor was the most abundant compound in both years, with concentrations ranging from 65.52% to 84.77% in 2023 and 25.37% to 60.57% in 2024 (Table S1). The mean concentrations were 3.78 ± 0.24 mg g FW^−^^1^ and 2.20 ± 0.28 mg g FW^−^^1^, respectively. The high camphor content, coupled with the absence of carvone, permitted the classification of the studied costmary plants to the “camphor” chemotype [32,36,41,42,43]. This dominance suggested a genetically stable chemotype, yet its quantitative variation between years might reflect climatic or phenological influences on the downstream biosynthesis of camphor via the mevalonate pathways.

Borneol, a terpene with known insect-repellent properties, was found at concentrations ranging from 5.41% to 17.88% in 2023 (mean: 592.60 ± 79.27 µg g FW^−^^1^) and 2.18% to 3.80% in 2024 (mean: 140.09 ± 18.18 µg g FW^−^^1^).

1,8-cineol ranged from 1.39% to 5.65% in 2023 and from 2.78% to 7.43% in 2024 with mean concentrations of 154.04 ± 22.12 µg g FW^−^^1^ and 194.99 ± 26.48 µg g FW^−^^1^, respectively. The increase in 1,8-cineol and decrease in borneol in 2024 could reflect competitive flux through the terpene biosynthetic network, suggesting a shift in enzyme activity that is possibly modulated via year-specific stress factors such as drought or temperature.

Terpinen-4-ol, a biologically active monoterpene tertiary alcohol important for its wide-ranging biological activities [44], showed a percentage range of 0.97% to 6.92% in 2023 (mean: 9.79 ± 1.19 µg g FW^−1^) and 1.92% to 3.73% in 2024 (mean: 67.38 ± 43.31 µg g FW^−1^). These variations might suggest a regulatory shift at the gene expression level of terpene synthases influenced by microbial root interactions or soil nutrient dynamics under compost addition.

Although α- and β-thujone were not influenced by treatments, they exhibited noteworthy characteristics in costmary. α-thujone ranged from 0% to 3.42% in 2023, and from 0.95 to 2.52% in 2024, differing from previous findings by [30,38,45], who reported percentages of 13.75%, 4.68%, and 11.4%, respectively. The mean concentration of α-thujone was 68.73 ± 15.10 µg g FW^−1^ in 2023 and 63.56 ± 13.47 µg g FW^−1^ in 2024. β-thujone was absent in 2023 but appeared in 2024 at levels ranging from 15.61% to 23.00%, with a mean concentration of 839.58 ± 123.81 µg g FW^−1^, also differing from the aforementioned studies. Such interannual discrepancies may reflect the delayed activation of biosynthetic routes, likely triggered via cumulative environmental signals or maturation stage, potentially coupled with gene–environment interactions specific to the camphor chemotype. These differences may be attributed to the chemotype, as the plants analyzed in those studies belonged to the “carvone” chemotype, whereas our study examined a “camphor” chemotype [32]. Further investigation is required to determine whether thujone levels are influenced by plant accessions or environmental conditions. Formisano et al. [46] described *T. vulgare* subsp. *siculum* as a thujone chemotype, suggesting that the high thujone content could explain the high sun exposure of plants. The high concentration of thujone in our plants in 2024 could have been influenced by high sun exposure. In our study, elevated thujone levels in 2024 may be linked to increased solar radiation or thermal stress, potentially enhancing the activity of thujone synthase enzymes. Although thujone has antibacterial, pesticidal, and insecticidal effects, the use of costmary essential oils rich in α-thujone and β-thujone should be undertaken carefully [30,32] since EMA (European Medicines Agency) [47] and the EC (European Commission) [48] recommend a maximum intake of thujone of 3–7 mg day^−^^1^ (EMA, 2012).

Regarding the reported toxicity of thujone, which has been linked to absinthism, studies indicate that thujone plays a minimal or negligible role in this condition [49,50]. According to these studies, the primary cause of absinthism symptoms is high alcohol consumption, rather than thujone. Moreover, the neurotoxic effects of thujone are primarily connected to the GABA-gated chloride channel, where α-thujone is approximately two to three times more potent as a modulator than β-thujone.

Lastly, three compounds—α- and β-phellandrene and (−)-myrtenol—were identified for the first time in costmary accessions cultivated in Italy. Some authors reported their presence in accessions from Turkey [51]. α-phellandrene ranged from 0.08% to 0.19% in 2023 (mean: 6.92 ± 0.77 µg g FW^−^^1^) and from 1.11% to 2.76% in 2024 (mean concentration of 69.17 ± 14.51 µg g FW^−^^1^; Table 2). β-phellandrene was found at 0.14% to 0.34% in 2023 (mean: 11.83 ± 1.29 µg g FW^−1^) and 1.69% to 4.45% in 2024 (mean: 112.26 ± 18.59 µg g FW^−1^). The emergence of phellandrenes in 2024 might point to a threshold-dependent expression pattern or microbial signaling effects, activating minor pathways in terpene metabolism.

Finally, myrtenol, a bicyclic monoterpene alcohol with a pleasant scent, was detected at 0.73% to 1.01% in 2023 (mean: 45.58 ± 3.80 µg g FW^−1^) and 0.36% to 0.46% in 2024 (mean: 18.07 ± 2.59 µg g FW^−1^). It is a widely used aroma compound in food and cosmetics, with a recognized safety status [52,53]. It has been reported in several species, including *Rhodiola rosea* L., *Aegle marvels* (L.) Correa, and *Myrtus communis* L., and even in Lithuanian *Tanacetum vulgare* L. leaves [54]. Its pharmacological effects include anti-inflammatory [55], antioxidant [56], anxiolytic [57], anti-microbial [58], anti-nociception [59], and gastroprotective activities [60], largely due to its regulatory effects on inflammatory cytokines, antioxidant enzymes, apoptosis, and GABA modulation. Its detection supports the potential for regional chemotypic differentiation, with implications for the valorization of Italian costmary in pharmaceutical and nutraceutical applications.

**Figure 3:**
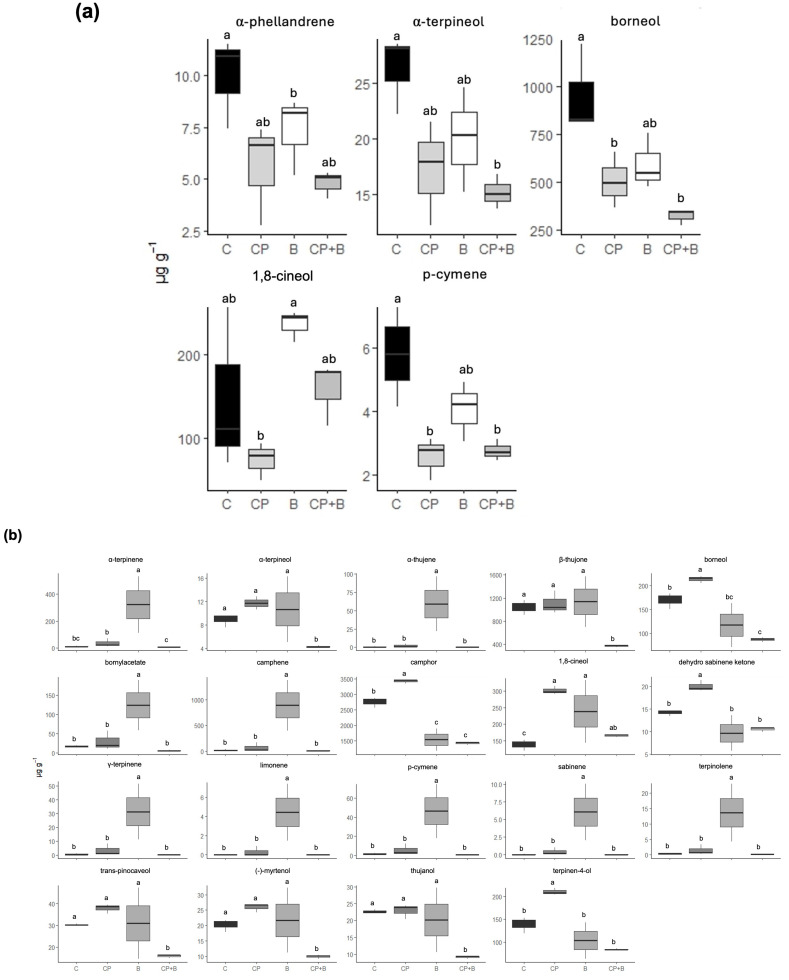
Box plots of the single compounds that have been found to be statistically different in 2023 (**a**) and in 2024 (**b**) in *Tanacetum balsamita* L. among treatments. C, control or no treatments; CP, compost from organic waste; B, bioinoculant; CP + B, compost and bioinoculant as the sum of CP and B treatments. Different letters within the same compound indicate significant differences according to Tukey’s HSD multiple-range test (*p* = 0.05).

Regarding the statistical differences in the single compounds among the treatments, significant variation was observed in 2023 for few compounds, namely α-phellandrene, α-terpineol, borneol, 1,8-cineol, and p-cymene (Figure 3a). These results suggested that, even in the absence of broad treatment effects across all compounds, certain metabolites are responsive to specific agronomic inputs under particular environmental conditions. In 2024, the number of compounds with statistically significant variation increased markedly, with nineteen compounds affected by treatments (Figure 3b). This temporal shift indicates that environmental factors prevailing in the second season may have interacted with the treatments to modulate metabolic pathways more strongly than in the first year.

**Figure 4:**
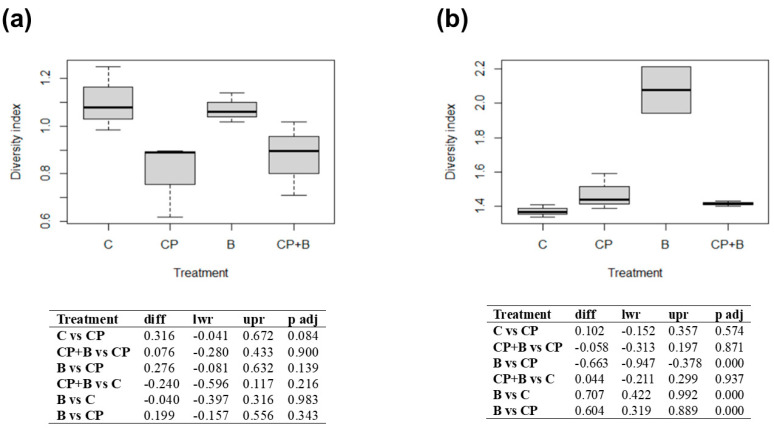
Diversity index for 2023 (**a**) and 2024 (**b**). C, control or no treatments; CP, compost from organic waste; B, bioinoculant; CP + B, compost and bioinoculant as the sum of CP and B treatments.

To better understand the breadth and distribution of essential oil constituents within a given treatment, diversity indices have been calculated since they are essential tools in ecological and environmental studies to offer insight into both richness (the number of compounds) and evenness (how equally means are distributed among those compounds). When the index is equal to 0, it reflects a total dominance by a single compound, indicating low biochemical complexity. Conversely, higher index values reflect more balanced metabolic profiles, suggesting a broader activation of biosynthetic routes. Overall, the diversity indices in both 2023 and 2024 (Figure 4) were above 0 for all treatments, indicating that there were many different compounds that were also distributed relatively evenly. In 2023, although some variation in diversity index values was noted—particularly between C and CP treatments with a *p*-value of 0.084—none of the comparisons are statistically significant at the 0.05 level (Figure 4a), indicating that treatments did not substantially alter the metabolic diversity of essential oils in the first growing season. However, in 2024 (Figure 4b), diversity indices increased overall (all > 1), with B treatment exhibiting significantly higher diversity compared to the others (*p* < 0.0005). This suggests that bioinoculants may stimulate a broader spectrum of secondary metabolic pathways, potentially through plant–microbe interactions such as induced systemic resistance or the microbial enhancement of micronutrient availability, which may, in turn, affect terpene biosynthesis.

The results presented in Table 2 provide an overview of the significance of the three key experimental factors—the treatment (likely referring to different experimental conditions or interventions), the year (indicating variations across different years or time points), and the treatment × year interaction (assessing whether the effect of treatment depends on the specific year)—for each compound. This multifactorial approach allows a mechanistic interpretation of how both agronomic inputs and seasonal variation modulate essential oil profiles [61].

The treatment factor had a minimal influence on the compounds overall, as indicated by the predominance of “n.s.” (not significant) entries for treatment across the majority of compounds. This suggests that the applied treatments did not significantly affect the results for many of the compounds, and the variability observed across these compounds is more likely due to other factors, such as the year or the treatment × year interaction. For example, compounds like α-thujene, camphene, α-terpinene, limonene, p-cymene, terpinolene, α-thujone, bornylacetate, γ-terpinene, terpinenen 4-ol, myrtenol, and some others showed no significant treatment effects. This suggests that these constituents are more strongly regulated by intrinsic plant genetics or environmental cues, rather than external agronomic inputs. However, notable exceptions emerged, such as camphor and dehydro sabinene ketone, which showed significant effects for treatment in 2024. Camphor was significantly affected by treatment at *p* ≤ 0.01, and dehydro sabinene ketone at *p* ≤ 0.001, suggesting that, in these cases, the treatment strongly influenced key steps in the terpene biosynthetic pathway—such as hydroxylation or ketone formation—potentially via hormonal modulation or enhanced nutrient uptake. These findings highlight that certain core or signature compounds may serve as biomarkers for the treatment response. On the contrary, the year factor appeared to have a greater influence on the outcomes of many of the compounds when compared to treatment effects, with numerous compounds showing a highly significant year effect. For instance, sabinene, α-phellandrene, β-phellandrene, trans-pinocarveol, trans-verbenol, α-terpineol, β-thujone, camphor, dehydro sabinene ketone, borneol, and several other compounds showed significant results for the year factor at varying levels of significance (e.g., *p* ≤ 0.05, *p* ≤ 0.01, *p* ≤ 0.001). This finding underscores the dominant role of environmental variability—including temperature, rainfall, photoperiod, and plant ontogeny—in shaping essential oil composition. It is plausible that these conditions affect the expression or activity of terpene synthases and other enzymes involved in oil biosynthesis, leading to substantial interannual differences.

For example, sabinene showed a significant effect for the year factor at *p* ≤ 0.05, suggesting that there were notable differences in the levels or behavior of sabinene across different years, even though treatment and treatment × year interactions were not significant. Similarly, α-phellandrene and β-phellandrene exhibited even more significant effects (*p* ≤ 0.01 for α-phellandrene and *p* ≤ 0.01 for β-phellandrene), pointing to a strong year-dependent influence on these compounds. This finding may suggest that external environmental conditions or plant senescence that varied across years could have played a role in altering the composition or concentration of these compounds. Similarly, β-thujone was significantly affected by the year factor at *p* ≤ 0.01, which could imply that the compound’s characteristics were influenced by temporal factors such as climate, temperature, or other yearly environmental changes. Compounds like camphor and dehydro sabinene ketone demonstrated even stronger responses to the year factor, exhibiting both significant year effects at *p* ≤ 0.001. This suggests that these compounds experience profound year-to-year variability that may be attributed to changes in environmental conditions/seasonal effects that could influence compound production or accumulation. The fact that the year is a major factor in the variation of these compounds emphasizes the importance of temporal consistency when interpreting experimental data, as year-to-year differences could overshadow other factors like treatment or interaction, as well as how different treatments may influence these compounds.

The treatment × year interaction further elucidated the complex interplay between agronomic practices and environmental conditions. For most compounds, the treatment × year interaction was not significant, as indicated by “n.s.” in the corresponding cells in Table 2. This suggests that, for most compounds, the differences due to treatment were relatively consistent across years, or that there was no clear interaction between these two factors. However, there were notable exceptions where the treatment × year interaction exhibited significant interactions, i.e., for 1,8-cineol, borneol, terpinen-4-ol, dehydro sabinene ketone, 3-thujanol, and α-terpineol. 1,8-cineol exhibited a significant interaction at *p* ≤ 0.01, indicating that its accumulation depends not only on the treatment or the season alone but also on their combination. This could suggest that the compound responds differently to the treatments in different years, possibly due to fluctuating environmental factors or other external conditions that affect its production or stability. Similarly, borneol showed a significant treatment × year interaction, suggesting that its levels or characteristics depend on both the treatment condition and the specific year and highlighting the importance of considering the combined effect of both factors in understanding the variability of this compound. Highly significant interactions (e.g., *p* ≤ 0.05, *p* ≤ 0.001) for 4-ol-terpinen, dehydro sabinene ketone, 3-thujanol, and α-terpineol, supported this idea, pointing to plasticity in the biosynthetic network, where enzyme activities or intermediate fluxes are fine-tuned via the external environment. This mechanistic insight suggests that designing effective agronomic strategies for modulating essential oil profiles must account not only for the inputs themselves but also for their interaction with the dynamic growing environment. These results highlighted the complexity of how experimental factors interacted and underscored the need for careful experimental design and analysis to tease apart the individual and combined effects of treatment and temporal variability. They demonstrated that, while treatment alone may influence specific oil constituents, the predominant source of variation lies in the year and in the treatment × year interaction, emphasizing the importance of conducting multi-season trials. Understanding how seasonal variables interact with bio-based treatments will be essential for optimizing essential oil quality and standardization in aromatic species like *Tanacetum balsamita* L.

## 3. Conclusions

This study has presented a comprehensive analysis of the chemical composition of essential oils extracted from fresh leaves of *T. balsamita* L. and the effects of sustainable agronomic treatments on specific compounds. Based on the dominant presence of camphor, plants were classified as belonging to the “camphor” chemotype. Three compounds—α- and ß-phellandrene and (−)myrtenol—were identified for the first time in Italian-grown costmary. Compost and bioinoculants enhanced the concentration of several key components of essential oil from costmary, such as camphor, borneol, terpinen-4-ol, α-terpineol, dehydro sabinene ketone, and 3-thujanol. While treatment effects were significant regarding the increase in the concentration of some compounds, the interaction between treatment and year was significant for a few compounds (borneol, terpinen-4-ol, dehydro sabinene ketone, 1,8-cineol, and 3-thujanol), suggesting that, in most cases, the treatment effect did not vary significantly across different years. These findings emphasize the importance of considering temporal variability in essential oil research also by industries and growers, particularly in long-term studies or in breeding studies that span multiple years. Despite being limited to two seasons and one site, this study provides a solid basis for future long-term and multi-environment trials focused on sustainable aromatic plant production.

## 4. Materials and Methods

### 4.1. Experimental Set Up

Plants of costmary were grown in Florence (Italy) at the Giardino Santa Maria Novella (43°49.062′ N, 11°14.200′ E, at 95 m. a. s. l.) during 2023 and 2024 growing seasons. In February 2023, nursery-propagated plants at the three-leaf stage, purchased from Quadrilatero SS Società Agricola (Albegna, Italy), were transplanted into the field. These same individuals, which propagate via rhizomes, were not replaced but only cut back after the first season, allowing for regrowth from the original planting material in 2024. Leaf samples were harvested at the beginning of July of both years prior to flowering. As such, the second-year growth originated from the same seed source and clonal individuals used in 2023, ensuring consistency in plant material across both years of the study. A soil analysis indicated that plants were grown in a silty clay loam soil with a nearly neutral pH, 1.5% carbon, and a C/N ratio of 8.6 in 2023 and 2024. Meteorological data for the growing seasons are provided in Table 3.

Traditional organic cultivation practices—such as fertilization with manure prior to transplanting in March of each experimental year, and the absence of herbicide and insecticide treatments—were uniformly applied across all experimental plots (both treated and untreated). These baseline practices were then compared with innovative approaches involving the use of bioinoculants, either alone or in combination with compost. A randomized block design (plot of 5.76 m^2^ each) with three replicates per treatment was adopted. Four treatments were tested: (i) control (C), no treatment; (ii) organic waste compost (CP) by Sienaambiente SpA (Siena, Italy) applied at 6 t ha^−^^1^ (equivalent to 3.6 kg m^−^^2^); (iii) bioinoculant (B), application of Bioseed (Unmaco Srl, Cerano, Italy), containing 5% mycorrhizae and rhizobacteria at 1010 CFU g^−^^1^, at 2 ‰ (equivalent to 2 L plant^−^^1^); (iv) compost and bioinoculant (CP + B), combination of compost and bioinoculant treatment. Compost treatment was applied once, in April 2023, whereas the bioinoculant was administered in both April 2023 and April 2024.

### 4.2. Tanacetum balsamita L. Harvest, Essential Oil Extraction, and GC-MS Analysis

Plants were harvested in June 2023 and June 2024 before flowering. Immediately after harvest in both years, fresh leaves were extracted using steam flow distillation (InHerba, Stallavena, Italy) for 1 h. Approximately 800 g of fresh leaves from each plot were distilled in 3 L of distilled water. The resulting essential oil (2 mL) was collected and immediately transferred into sealed vials and stored refrigerated until analysis. The essential oils were analyzed using gas chromatography–mass spectrometry GC-MS as soon as possible after extraction to ensure sample integrity.

Analyses were performed on an Agilent 7820A (Agilent Technologies, Santa Clara, CA, USA) gas chromatograph coupled with an Agilent 5977E mass spectrometer with electronic ionization and a single quadrupole. Sample injection was automated using a Gerstel MPS2 XL autosampler. The GC methodology is described below:

- Column: Agilent DB-Wax UI polar capillary column (160 m length, i.d. 0.250 mm, thickness 0.5 μm);
- Carrier gas: Helium at 1.2 mL min^−^^1^;
- Oven temperature program: initial = 40 °C; ramp = 5 °C min^−1^ to 200 °C, then 10 °C min^−^^1^ to 240 °C;
- Injection: splitless, 1 µL volume, 1 min splitless time;
- Injection temperature: 250 °C;
- Mass spectrometer settings: ionization energy = 70 eV, scan range = 29–330 amu, scan rate = 4.5 scans sec^−^^1^.

The total run time was 35 min. Compounds were identified by comparing mass spectra with the NIST Mass Spectral Library. The relative concentration of each identified compound was calculated as the peak area percentage of the total identified peaks. Tridecane was used as an internal standard for normalization, and standards were used for the quantification. Each compound was quantified via a six-point calibration (concentration range 1–200 ppm; R^2^ = 0.998). The concentration of individual compounds was expressed in ppm based on GC-MS analysis, and the quantities were calculated as micrograms per gram of fresh weight (µg g^−^^1^ FW).

### 4.3. Statistical Analysis

Two types of analysis were carried out. Firstly, normality of data distribution was assessed using the Shapiro–Wilk test and homogeneity of variances with Levene’s test. For compounds not detected in some samples, data were carefully handled by excluding non-detected values from the parametric analysis to avoid violating test assumptions. In cases where data did not meet these criteria, non-parametric tests were considered. Then, a one-way analysis of variance (ANOVA) was conducted to evaluate treatment effects on compound concentrations. The ANOVA model was specified as follows:

concentration ∼ treatment

A significance level of *α* = 0.05 was applied to all tests. If the ANOVA showed significant effects, Tukey’s honestly significant difference (HSD) test was used for pairwise comparisons between treatments.

Secondly, another analysis aimed to assess the differences in diversity of costmary’s essential oils derived from plants treated with the four different agronomic practices. The dataset comprised diversity Shannon–Wiener indices calculated for each treatment group, categorized into the four treatments: C, CP, B, and CP + B. A one-way ANOVA was performed to assess treatment effects on diversity indices, followed by Tukey’s HSD test for post hoc pairwise comparisons when significant differences were found. A significance level of α = 0.05 was used for all statistical tests.

Finally, a two-way repeated measures ANOVA was used to analyze the whole dataset (2023 + 2024) in order to determine whether there were significant differences among groups over time and across different treatments while accounting for individual variability. The year was treated as the repeated measure factor, while plot was considered the blocking factor to account for variability between experimental units. The Student–Newman–Keuls (SNK) test was applied as a mean comparison test to describe the differences between the main factors.

The statistical analysis was conducted using R software (version 4.2.2) with the following packages: ggplot2, agricolae, reshape2, dplyr, and multcompView.

## Figures and Tables

**Table 1:** Volatile compound composition (µg g^−1^ FW) in essential oils of costmary plants cultivated with four different agronomic practices in 2023 and 2024. n.d., not detected.

		2023								2024							
		C		B		CP		C + I		C		B		CP		C + I	
		Min	Max	Min	Max	Min	Max	Min	Max	Min	Max	Min	Max	Min	Max	Min	Max
		(µg g^−1^ FW)		(µg g^−1^ FW)		(µg g^−1^ FW)		(µg g^−1^ FW)		(µg g^−1^ FW)		(µg g^−1^ FW)		(µg g^−1^ FW)		(µg g^−1^ FW)	
1	α-thujene	3.3	8.2	3.4	7.2	2.3	4.3	2.5	5.9	0.1	1.1	0.1	97.0	0.2	4.9	0.2	0.4
2	camphene	1.2	15.9	1.9	3.0	0.3	0.4	0.5	1.0	4.9	36.3	2.5	1380.1	9.8	172.1	9.1	13.3
3	Sabinene	6.0	14.2	6.4	13.3	3.9	7.5	4.3	10.2	n.d.	0.1	n.d.	10.0	0.1	1.1	n.d.	0.1
4	α-phellandrene	7.4	11.5	5.2	8.7	2.8	7.4	4.0	5.3	60.4	67.0	14.0	206.7	83.4	94.3	30.5	33.7
5	α-terpinene	8.6	17.3	7.5	14.6	5.3	9.8	5.8	10.8	8.1	17.1	2.1	532.0	13.9	70.3	6.3	8.4
6	limonene	1.3	2.9	1.1	2.1	1.0	1.5	0.9	1.9	n.d.		n.d.		n.d.		n.d.	
7	β-phellandrene	12.0	19.6	9.3	14.6	4.9	12.2	7.1	9.8	94.0	109.0	21.0	333.0	141.0	157.0	51.0	55.0
8	1.8-cineol	70.7	266	214.1	248.1	49.5	93.9	114.0	181.0	119.0	153.0	43.0	334.0	292.0	316.0	161.0	169.0
9	γ-terpinene	5.0	11.9	5.1	11.4	3.3	6.5	3.5	8.7	0.2	1.2	0.1	51.5	0.6	8.2	0.3	0.5
10	p-cymene	4.1	7.5	3.0	4.9	1.8	3.1	2.5	3.1	1.0	2.8	0.3	74.7	1.8	12.2	0.8	1.1
11	terpinolene	3.0	6.6	2.7	6.1	1.8	3.7	1.9	4.6	n.d.	0.6	0.1	23.0	0.4	3.3	0.2	0.3
12	α-thujone	40.2	165	59.8	162.7	9.8	47.3	n.d.	107.0	61.1	81.2	15.4	188.7	62.2	87.3	21.4	24.1
13	β-thujone	n.d.		n.d.		n.d.		n.d.		911.4	1160.0	233.5	1575.0	952.3	1324.0	352.6	383.8
14	Camphor	3191.0	5828.0	3110.0	4563.0	3592.0	4163.0	2470.0	4259.0	2567.0	2882.0	806.0	1897.0	3349.0	3461.0	1363.0	1449.0
15	bornylacetate	67.2	202.0	36.8	86.0	31.7	52.9	32.0	60.1	14.9	20.3	4.9	189.0	11.3	58.1	5.7	7.8
16	terpinen 4-ol	283.0	388.0	183.0	342.0	0.0	257.0	41.2	190.0	119.5	153.2	31.9	143.4	204	218.5	83.2	86.2
17	dehydro sabinene ketone	n.d.		n.d.		n.d.		n.d.		0.27	0.34	0.18	0.30	13.5	14.6	3.7	13.6
18	3-thujanol	n.d.		n.d.		n.d.		n.d.		0.44	0.52	0.35	0.40	22.4	23.3	4.8	29.7
19	trans-Pinocarveol	n.d.		n.d.		n.d.		n.d.		0.60	0.70	0.48	0.65	29.9	30.9	8.2	47.3
20	trans-Verbenol	n.d.		n.d.		n.d.		n.d.		0.26	0.28	0.28	0.60	12.1	13.1	3.5	44.7
21	α-terpinenol	22.2	28.5	15.2	24.6	12.2	21.5	13.8	16.9	7.5	9.6	1.9	16.3	10.6	12.9	4.2	4.5
22	Borneol	813.0	1222.0	478.0	753.0	365.0	654.0	272.0	349.0	150.8	182.8	38.3	163.2	205.1	219.2	81.4	90.8
23	verbenone	6.6	12.1	8.7	3.5	6.0	11.9	7.5	10.5	n.d.		n.d.		n.d.		n.d.	
24	Carvone	17.0	30.8	13.7	16.8	13.9	25.5	12.6	17.1	n.d.		n.d.		n.d.		n.d.	
25	(−)-myrtenol	44.5	72.7	34.1	59.5	28.7	48.6	24.1	50.4	17.8	21.7	5.7	32.2	24.2	26.7	9.2	10.3

**Table 2:** Results of the Student–Newman–Keuls (SNK) test as a post hoc comparison to examine differences between the main factors in a two-way repeated measures ANOVA. n.s. = not significant, or *, **, *** = significant at *p* ≤ 0.05, 0.01, or 0.001, respectively.

	Compound	Factor		
		Treatment	Year	Treatment × Year
1	α-thujene	n.s.	n.s.	n.s.
2	camphene	n.s.	n.s.	n.s.
3	sabinene	n.s.	*	n.s.
4	α-phellandrene	n.s.	**	n.s.
5	α-terpinene	n.s.	n.s.	n.s.
6	limonene	n.s.	n.s.	n.s.
7	β-phellandrene	n.s.	**	n.s.
8	1,8-cineol	n.s.	n.s.	**
9	γ-terpinene	n.s.	n.s.	n.s.
10	p-cymene	n.s.	n.s.	n.s.
11	Terpinolene	n.s.	n.s.	n.s.
12	α-thujone	n.s.	n.s.	n.s.
13	β-thujone	n.s.	**	n.s.
14	camphor	**	***	n.s.
15	bornylacetate	n.s.	n.s.	n.s.
16	terpinen 4-ol	*	*	*
17	dehydro sabinene ketone	***	***	***
18	3-thujanol	*	***	*
19	trans-pinocarveol	n.s.	***	n.s.
20	trans-verbenol	n.s.	***	n.s.
21	α-terpineol	*	***	n.s.
22	borneol	***	***	**
23	Verbenone	n.s.	***	n.s.
24	Carvone	n.s.	***	n.s.
25	(−)-myrtenol	n.s.	***	n.s.

**Table 3:** Meteorological data during *Tanacetum balsamita* L.’s growth season in 2023 and 2024.

	Mean Temperature (°C)	T Min (°C)	T Max (°C)	Mean Humidity (%)	Mean Wind Speed (km h^−1^)	Rain (Days)	Hail (Days)	Summer Storms (Days)	Fog (Days)
**2023**									
May	18.7	12.9	23.7	67.1	9.3	15	0	5	2
June	23.3	17.1	29.6	65.8	7.1	14	0	12	0
**2024**									
May	18.7	13.1	24.2	69.0	3.7	11	0	2	1
June	22.4	16.2	28.3	65.2	0.0	7	0	4	0

## Data Availability

The original contributions presented in this study are included in the article/Supplementary Material. Further inquiries can be directed to the corresponding author.

## Supplementary Materials

The following supporting information can be downloaded at: https://www.mdpi.com/article/10.3390/plants14152406/s1.
