# Supplementary material for: Sustainable Agronomical Practices Affect Essential Oil Composition of Tanacetum balsamita L
Source: Plants (Basel). 2025 Aug 3;14(15):2406. doi: 10.3390/plants14152406 (PMC12349446; doi:10.3390/plants14152406)
Supplement: Supplementary file 1 [file plants-14-02406-s001.zip › plants-3685427-supplementary.pdf]

Table S1. Volatile compound composition (%) in essential oils of costmary plants cultivated with four different agronomic practices in 2023 and 2024.

	Compound	2023							
		C		B		CP		CP+B	
		Min (%)	Max (%)	Min (%)	Max (%)	Min (%)	Max (%)	Min (%)	Max (%)
1	α-thujene	0.07	0.11	0.06	0.15	0.06	0.15	0.06	0.14
2	camphene	0.02	0.28	0.04	0.06	0.04	0.06	0.01	0.02
3	sabinene	0.13	0.21	0.11	0.28	0.11	0.28	0.11	0.24
4	α-phellandrene	0.15	0.19	0.12	0.17	0.12	0.17	0.08	0.15
5	α-terpinene	0.19	0.28	0.15	0.31	0.15	0.31	0.12	0.25
6	limonene	0.03	0.04	0.02	0.04	0.02	0.04	0.02	0.05
7	β-phellandrene	0.25	0.34	0.22	0.31	0.22	0.31	0.14	0.25
8	cineol	1.39	4.74	3.46	5.65	3.46	5.65	3.46	4.24
9	γ-terpinene	0.11	0.21	0.09	0.24	0.09	0.24	0.08	0.20
10	p-cymene	0.07	0.13	0.07	0.10	0.07	0.10	0.05	0.08
11	terpinolene	0.06	0.11	0.05	0.13	0.05	0.13	0.05	0.11
12	α-thujone	0.84	2.94	0.97	3.42	0.97	3.42	0.00	2.51
13	β-thujone	n.d.		n.d.		n.d.		n.d.	
14	camphor	65.52	73.27	71.69	73.82	71.69	73.82	74.67	84.77
15	bornylacetate	1.45	2.54	0.85	1.81	0.85	1.81	0.97	1.41
16	terpinen 4-ol	3.55	6.92	4.03	5.53	4.03	5.53	0.97	5.74
17	dehydro sabinene ketone	n.d.		n.d.		n.d.		n.d.	
18	3-thujanol	n.d.		n.d.		n.d.		n.d.	
19	trans-pinocarveol	n.d.		n.d.		n.d.		n.d.	
20	trans-verbenol	n.d.		n.d.		n.d.		n.d.	
21	α-terpineol	0.36	0.50	0.35	0.43	0.35	0.43	0.34	0.42
22	borneol	14.48	17.88	10.05	12.64	10.05	12.64	5.41	10.56
23	verbenone	0.14	0.18	0.20	0.22	0.20	0.22	0.21	0.24
24	carvone	0.34	0.39	0.27	0.35	0.27	0.35	0.32	0.40
25	(-)-myrtenol	0.90	0.96	0.79	1.01	0.79	1.01	0.73	1.00

	Compound	2024							
		C		B		CP		CP+B	
		Min (%)	Max (%)	Min (%)	Max (%)	Min (%)	Max (%)	Min (%)	Max (%)
1	α-thujene	0.00	0.02	0.00	1.30	0.00	0.08	0.01	0.02
2	camphene	0.11	0.72	0.20	18.45	0.18	2.69	0.39	0.55
3	sabinene	0.00	0.00	0.00	0.13	0.00	0.02	0.00	0.00
4	α-phellandrene	1.28	1.41	1.11	2.76	1.48	1.56	1.34	1.40
5	α-terpinene	0.19	0.34	0.17	7.12	0.25	1.10	0.26	0.35
6	limonene	0.00	0.00	0.00	0.10	0.00	0.01	0.00	0.00
7	β-phellandrene	2.17	2.19	1.69	4.45	2.43	2.55	2.16	2.28
8	cineol	2.78	3.03	3.44	4.69	4.57	5.40	6.72	7.43
9	γ-terpinene	0.01	0.02	0.01	0.69	0.01	0.13	0.01	0.02
10	p-cymene	0.02	0.06	0.03	1.00	0.03	0.19	0.03	0.05
11	terpinolene	0.00	0.01	0.00	0.31	0.01	0.05	0.01	0.01
12	α-thujone	1.43	1.61	1.22	2.52	1.12	1.37	0.95	1.01
13	β-thujone	21.28	23.00	18.55	22.90	17.21	20.73	15.61	16.03
14	camphor	57.16	59.94	25.37	64.02	54.08	60.50	59.93	60.57
15	bornylacetate	0.35	0.40	0.39	2.53	0.20	0.91	0.24	0.32
16	terpinen 4-ol	2.79	3.04	1.92	2.53	3.26	3.73	3.48	3.69
17	dehydro sabinene ketone	0.27	0.34	0.18	0.30	0.30	0.37	0.44	0.45
18	3-thujanol	0.44	0.52	0.35	0.40	0.37	0.41	0.38	0.39
19	trans-pinocarveol	0.60	0.70	0.48	0.65	0.61	0.67	0.66	0.69
20	trans-verbenol	0.26	0.28	0.28	0.60	0.30	0.35	0.29	0.33
21	α-terpineol	0.18	0.20	0.15	0.22	0.19	0.20	0.17	0.19
22	borneol	3.52	3.65	2.18	3.04	3.38	3.74	3.60	3.80
23	verbenone	n.d.		n.d.		n.d.		n.d.	
24	carvone	n.d.		n.d.		n.d.		n.d.	
25	(-)-myrtenol	0.42	0.44	0.36	0.46	0.42	0.46	0.41	0.43
